# Potential Effects of Rift Valley Fever in the United States

**DOI:** 10.3201/eid1708.101088

**Published:** 2011-08

**Authors:** David M. Hartley, Jennifer L. Rinderknecht, Terry L. Nipp, Neville P. Clarke, Gary D. Snowder, the National Center for Foreign Animal and Zoonotic Disease Defense Advisory Group on Rift Valley Fever

**Affiliations:** Author Affiliations: Georgetown University, Washington, DC, USA (D.M. Hartley);; National Institutes of Health, Bethesda, MD, USA (D.M. Hartley);; National Center for Foreign Animal and Zoonotic Disease Defense, College Station, Texas, USA (T. L. Nipp, J. L. Rinderknecht, G. D. Snowder);; Texas A&M University, College Station (N.P. Clarke)

**Keywords:** Rift Valley fever, disease models, animal, insect vectors, economic models, epidemiology, zoonoses, mosquitoes, arboviruses, viruses, online report

## Workshop Summary

Rift Valley fever virus (RVFV) has been the cause of disease outbreaks throughout Africa and the Arabian Peninsula, and the infection often results in heavy economic costs through loss of livestock. If RVFV, which is common to select agent lists of the US Department of Health and Human Services and the US Department of Agriculture, entered the United States, either by accidental or purposeful means, the effects could be substantial. A group of subject matter experts met in December 2009 to discuss potential implications of an introduction of RVF to the United States and review current modeling capabilities. This workshop followed a similar meeting held in April 2007. This report summarizes the 2 workshop proceedings. Discussions primarily highlighted gaps in current economic and epidemiologic RVF models as well as gaps in the overall epidemiology of the virus.

## Foreign Animal and Zoonotic Disease Defense Workshops

The potential effects on both human and animal health and the US economy from foreign animal and zoonotic disease (FAZD) threats is clear. The National Center for Foreign Animal and Zoonotic Disease Defense (FAZD Center) was founded in April 2004 to defend the United States from FAZD threats. One such threat is the accidental or deliberate introduction of Rift Valley fever virus (RVFV). This article reports on 2 FAZD Center workshops (in April 2007 and November 2009) that reviewed the status of US vulnerability to RVF and mitigation modeling and identified information and technology gaps. Workshop discussion centered on relevant biology and management strategies to include in RVF epidemiologic models, important effects to include in economic models of RVF consequences, and ways to integrate epidemic and economic models. Each major topic of discussion is summarized below.

## Disease Cycle

RVFV is transmitted to livestock and human hosts primarily by biting vectors and handling of infected animals by persons. In Africa and the Arabian Peninsula, competent vectors include numerous *Aedes* and *Culex* spp. mosquitoes. Recent competence studies have found that competent vectors in both genera exist in the United States (1). Field observations indicate that RVFV is vertically maintained during dry periods in the eggs of floodwater *Aedes* spp. mosquitoes, although transovarial transmission has not been observed in laboratory experiments (2). Workshop participants recommended that an earlier hypothesis regarding an RVFV sandfly/rodent cycle be studied further (3,4). Given the hardiness of *Aedes* spp. eggs, which have been found to remain viable in African soil for years, if RVFV were to be introduced into the United States, eradicating it may be difficult or impossible (5). Climatic, environmental, and ecologic factors such as the creation of larval mosquito habitat by above-normal amounts of rainfall are well-known antecedent events for African outbreaks, but the timing and interaction of these variables in the United States are unknown. Participants also noted that human morbidity and mortality rates in the United States may be different from those observed in rural Africa.

## Introduction Potential

At present, RVFV is confined to Africa and the Arabian Peninsula, but the pathogen poses a risk to nations outside the current area of distribution (6,7). In the United States, RVFV could be introduced and rapidly spread by mosquitoes, through livestock being traded, and by potentially unknown wildlife hosts, as in the case for West Nile virus (8). However, where or when RVFV is likely to enter the United States or what areas are at greatest risk for RVFV transmission is not known. US grassland prairie areas with rainfall and temperature comparable to disease-endemic areas in Africa and areas supporting beef cattle and sheep production may be at elevated risk. Air travel and cargo movement represent potential modes of entry because mosquitoes can be transported on planes and in cargo pods (9).

If RVFV is introduced into the United States, whether it would become enzootically established is not clear. Place of introduction and time of the year would influence whether RVFV enters an enzootic maintenance cycle. How quickly it is identified and which control measures are deployed would also influence whether it becomes enzootic. The particular hosts in the United States that are capable of becoming viremic will also influence the enzootic establishment of RVFV. Sheep, goats, and cattle can become viremic. Calves are susceptible to high viremia levels, but little information is known regarding the susceptibility of US adult cattle to wildlife. Expanded research to investigate the competence of potential wildlife hosts is of particular importance. If wildlife hosts (e.g., deer, antelope, elk) are capable of becoming viremic, controlling the spread of the virus would be difficult, if not impossible. If susceptible, populations of endangered species, such as bighorn sheep, may be further reduced.

Moreover, data are lacking on transovarial transmission rates and the ability of RVFV-competent mosquitoes to successfully overwinter with the virus; thus, determining whether the virus could survive during the winter season in various parts of the country is not possible. Analyses aimed at identifying regions likely to support transmission and establishment of RVFV should include the environmental variables that affect vector behavior, such as temperature (which affects the frequency of host contact, generation time, and the duration of the viral extrinsic incubation period), moisture (affects appetitive behavior), and the presence of breeding habitat (population presence/absence). Remote sensing of related variables over large areas may provide valuable data that can be used in mathematical models as well as maps of vector and host distributions in space and time. Antecedent ocean temperatures, in combination with real-time remotely sensed vegetation data, have been skillfully linked with outbreaks in Africa and may have predictive application in the United States should the virus become endemic (2,10).

## Exposure Pathways

In Africa, transmission associated with the rainy season begins when vertically infected floodwater adult *Aedes* spp. mosquitoes emerge from breeding sites and bite susceptible livestock, which in turn amplify the virus and enable horizontal transmission through multiple biting vectors. In addition to exposure to infected mosquitoes and (potentially) other biting insect vectors, cattle exhibit behavior such as eating, licking, and sniffing the placenta of an aborted fetus that may play a role in amplifying the disease. Handling of such tissues by humans has been found to be significantly associated with infection (11,12).

The frequency at which mosquitoes or other biting insects infected with RVFV bite humans and livestock is unknown. Conducting investigations, such as human-biting studies, may be difficult for several reasons. In general, sheep and cattle are hosts to large numbers of mosquitoes. Although only a small number of mosquitoes typically survive to infect humans, this fact could be critical for agricultural worker exposure. Because of the lack of proximity to viremic livestock experiencing a natural outbreak of RVF, incidence of human disease is likely to be lower in large cities, whereas areas in close proximity to livestock are likely to have a higher incidence. Recent observations in South Africa supported this hypothesis (13–15). However, if RVFV were intentionally introduced by aerosols, the disease incidence would be expected to be much higher in heavily populated areas. High viremia levels can develop in humans, and humans may be involved as amplifying hosts (16). Therefore, the possibility should be considered that infected travelers can introduce RVFV to residents of the areas they are visiting. The incidence of human infection and exposure in Africa may be not be generalizable to the United States, however, because of the differences in living conditions and proximity to livestock.

## Detection

Accidental or purposeful introduction would require a rapid response, but detection may be challenging. When farmers notice an increased incidence of sickness and abortion, veterinarians are generally contacted to determine the cause of the condition and initiate treatment. Because the symptoms of RVF in livestock can be confused with other pathogenic diseases that cause abortion (such as campylobacteriosis, infectious bovine rhinotracheitis, bluetongue, brucellosis, and trichomoniasis), misdiagnosis by the veterinarian can delay detection (17). Thus, US veterinarians should be trained to be suspicious of symptoms resembling RVF. Another problem is that many state laboratories are not allowed or equipped to culture RVFV and other BioSafety Level 4 agents. The diagnostic tests currently available must be performed in a laboratory. ELISAs will detect immunoglobulin M (for recent infections) or immunlgobulin G (to confirm RVF seroconversion after 1 week of infection). The PCR technique will enable the viral genome to be detected during the first week postinfection. Rapid field tests are not yet available. Workshop participants identified the need for better accessibility to these techniques and the importance of further development of such assays in preparing for a potential RVF event.

## Control Options

Options include vector control, vaccination of livestock and humans, culling of livestock, modification of livestock transportation, and modification of human behavior. Focusing control efforts on agricultural workers and human populations who live in proximity to livestock may be effective.

Interruption of epizootic/epidemic transmission would require the effective and wide use of adulticides to eliminate infected female mosquitoes for 10 days when amplifying host animals are viremic. Extensive larval control may have an effect by reducing subsequent mosquito populations but would not effectively interrupt ongoing widespread transmission. Determining whether adulticides alone (or in combination with larvicides) or larvicides alone are more effective for controlling RVFV transmission and emergence is essential. Different mosquito and biting insect species may be key vectors in different geographic areas. Because mosquito behavior varies from species to species and across regions, different control strategies may be appropriate in different areas. For example, in some areas, night-biting mosquitoes may play a large role in spreading the virus, while in other areas, day-biting mosquitoes are predominant; the corresponding controls may also be different. Human exposure may further vary, depending on income level and housing quality. Vectors resistant to pesticides can also be problematic and limit the array of control options.

Focusing human vaccination on groups at high risk, as opposed to mass vaccination, may be appropriate in areas with active transmission. Mass vaccination may be needed if aerosols are intentionally introduced into a heavily populated area. In either instance, whether to make vaccination compulsory or voluntary will likely be a major question for policy makers. Currently, no human or veterinary vaccines against RVFV are commercially available in the United States. Workshop participants also discussed the need for a vaccine that differentiates infected from vaccinated animals during pre-epidemic surveillance. Problems associated with the production of such an animal vaccine include the lack of a commercial partner and market and the fact that the national veterinary stockpile does not require it.

Mass culling of livestock suspected of RVFV infection is anticipated to be difficult for social and political reasons. The effect of culling on controlling spread is not well understood. Participants discussed the possibility that culling could make situations much worse because the decrease in living livestock hosts may drive mosquitoes to feed more frequently on wildlife and humans. Limiting the transportation of livestock may be important because livestock amplify the virus and can introduce it into new areas.

In addition, human behavior in response to public health agency guidance may not be easy to manage. During the West Nile virus invasion of the United States, for example, when the Centers for Disease Control and Prevention recommended that persons stay indoors, some took the recommendation to the extreme, while others ignored it. In general, participants advised that no control modality is likely to work well enough by itself and that combination strategies deserve analysis. The group also expressed the consensus view that vaccination can buy vital time to implement other strategies as well as protect first responders.

## Economic Dimensions

The economic effect of a potential RVFV outbreak in the United States was a topic of major discussion. Participants suggested that if RVFV becomes established before it is detected, achieving an effective response in the first year may be difficult. In such a scenario, an economic analysis could assume that 100% of pregnant and infected cattle will abort and that all calves <14 days of age within an affected area will die or be euthanized. Animals that die or are dying from the disease will not enter the food chain. Infected animals that survive are not expected to experience higher than normal abortion rates in subsequent years, in any case, because of acquired immunity. The effects of RVFV on milk production and marketing are unknown. Regulatory agencies (e.g., Occupational Safety and Health Administration) may not allow the infected cows to be milked; this decision would have a dramatic effect on milk production if a substantial number of cows were infected. Methods for handling and disposing of milk produced by infected commercial dairy cows have not been determined. Tests to determine RVFV presence in milk may be essential to minimize the potential effects of the disease on the dairy industry. The effect of RVFV on the meat industry and the supply of meat for human consumption could be devastating if meat is recalled and slaughter bans are enacted. The effects on the sheep and goat industries are not known; however, a shrinking and infrastructure-sensitive livestock industry, such as the sheep and goat industry, may not have the resilience to recover completely.

Several noteworthy costs are associated with a potential outbreak of RVFV. These costs stem from actions taken before and after the event. Preparation and preventive costs for an RVF event include heavy spending through vaccine and detection technology research, costs of stockpiling vaccines, costs of disease surveillance, and the cost of purchasing response and support equipment. Immediate costs after or during an initial outbreak include the loss of animals, disruptions in the supply chain, and mitigation efforts. Further economic loss may come from byproduct damages from quarantine and other control policies, lowered demand for feed and animal imports, and international trade restrictions imposed on US livestock. Human illness would result in costs from medical assistance and decreases in workforce productivity. The magnitude of these costs is unknown.

As stated above, international trade restrictions are likely to be imposed on US livestock, but the scale and consequences are unknown and are in need of modeling. Discussion also highlighted the need for response and support equipment, trained technical support for mosquito control, and facilities to be in place for diagnosis and treatment before any introduction of RVFV. The effects of not having materials, labor, and logistics ready for prompt action should also be modeled. In addition, major analysis is needed regarding the consequences of an introduction that does or does not become enzootically entrenched in the United States. Analyses are also necessary to highlight best-case versus worst-case consequences and ways to avoid costs. Economic analysis should also address the following major questions. How much effort should be spent on prevention? What is the likelihood that endemic status for RVFV could be avoided at reasonable cost? How much would human vulnerability cost?

## Important Gaps

Early detection and containment are key to interrupting the transmission of RVFV and preventing its establishment; however, the ability of current passive case surveillance to detect and rapidly report infection is questionable and must be improved. The lack of rapid diagnostic tools and field-deployable assays that can be standardized is another major gap. Understanding the ecologic properties that result in regions of efficient epidemic transmission and endemic focus is necessary for directing prevention and control measures. Identifying potential competent vectors, understanding their feeding preferences and biting rates, mapping their seasonal presence, and understanding the efficiency of pesticides against them are major needs. Whether wildlife hosts exist that may amplify the virus in a continuous enzootic cycle must also be determined. In addition, many unknowns remain concerning the structure and distribution of the US livestock transportation network. We must better understand the relative payoff between different control measures and the circumstances in which each approach or combinations of approaches are appropriate. The economic returns on potential control strategies must be investigated as well as the economic consequences of an RVF introduction.

## Implications for Modeling

Statistical models that predict RVF risk using ocean-based climate variables and subsequent rain-related surface water, remotely sensed by vegetation greenness, have been proposed (2). If RVFV is introduced into the United States, these models would have the added complexity of determining when, where, and how the introduction occurred. The performance of this approach is promising and recently was successful at forecasting disease activity in East-Central Africa (10). However, these models may be difficult to generalize to dissimilar ecologic areas; they do not include the effects of immunity or different animal–human interactions and cannot model the effects of control measures. Process-based mathematical models are needed to answer “what if” questions to clarify the dynamics of RVF as a function of temperature, vector and host population characteristics, and the effects of control programs. An initial effort has been described in the literature and is being generalized to investigate the control options described above and also the spatiotemporal spread caused by motion of vectors and transportation of livestock (18,19). At present, this model includes only livestock, *Aedes* spp.–like mosquito vectors, and *Culex* spp.–like mosquito vectors. Work is currently underway at the FAZD Center to expand this model into a more comprehensive model of the spatial spread of RVFV, incorporating vaccination, vector control, livestock movement controls, and other potential intervention measures, in addition to human health implications.

Filling the gaps identified above is key to unlocking the potential of models for providing robust guidance to decision makers. Participants concluded that to develop accurate, valid models, the following knowledge gaps need to be filled immediately: the economic implications of an RVFV introduction according to different scenarios; the potential for the virus to become endemic to some areas of the United States, but not in others; and the competency and control of US vectors.

## The Future

Workshop participants highlighted the importance of a holistic approach to RVFV defense. Vaccines, antiviral agents, detection/diagnostic tests, and universal platforms are needed to support disease detection and diagnosis. Reliable and validated modeling and analytic tools are essential to support informed policy and decision making in industry and government. In particular, modeling may play a vital role in evaluating the effects of control strategies, in estimating the potential geospatial spread of the disease, and in assessing economic consequences of a potential introduction of RVFV. Education and outreach are required to transfer knowledge to areas where it can have the strongest effect. The following components are critical: graduate programs to prepare future scientists; training of early responders; and conducting stakeholder workshops to identify current knowledge and data gaps, critique and review current research, and guide future endeavors. FAZD Center investigators and others are working to fill these needs. Future workshops are anticipated in which advances on these issues are reviewed.
